# Diverse functional evolution of serine decarboxylases: identification of two novel acetaldehyde synthases that uses hydrophobic amino acids as substrates

**DOI:** 10.1186/s12870-014-0247-x

**Published:** 2014-09-18

**Authors:** Michael P Torrens-Spence, Renee von Guggenberg, Michael Lazear, Haizhen Ding, Jianyong Li

**Affiliations:** Department of Biochemistry, Virginia Tech, Blacksburg, Virginia USA; Present address: Whitehead Institute for Biomedical Research, Cambridge, Massachusetts USA

**Keywords:** Type II PLP decarboxylases, Aromatic amino acid decarboxylase, Aromatic acetaldehyde synthases, Phenylacetaldehyde synthases, Serine decarboxylases

## Abstract

**Background:**

Type II pyridoxal 5′-phosphate decarboxylases are an important group of phylogenetically diverse enzymes involved in amino acid metabolism. Within plants, this group of enzymes is represented by aromatic amino acid decarboxylases, glutamate decarboxylases and serine decarboxylases. Additional evolutionary divergence of plant aromatic amino acid decarboxylases has resulted in further subcategories with distinct substrate specificities and enzymatic activities. Despite shared homology, no such evolutionary divergence has been characterized within glutamate decarboxylases or serine decarboxylases (SDC).

**Results:**

Comparative analysis of two previously characterized serine decarboxylase-like (SDC-like) enzymes demonstrates distinct substrate specificities despite their highly conserved primary sequence. The alternate substrate preference of these homologous SDC-like proteins indicated that functional divergence might have occurred with in SDC-like proteins. In an effort to identify additional SDC-like functional divergence, two uncharacterized SDC-like enzymes were recombinantly expressed and characterized.

**Conclusions:**

An extensive biochemical analysis of two serine decarboxylases-like recombinant proteins led to an interesting discovery; both proteins catalyze the formation of acetaldehyde derivatives from select hydrophobic amino acids substrates. Specifically, *Medicago truncatula* [GenBank: XP_003592128] and *Cicer arietinum* [GenBank: XP_004496485] catalyze the decarboxylation and oxidative deamination of phenylalanine, methionine, leucine and tryptophan to generate their corresponding acetaldehydes. The promiscuous aldehyde synthase activity of these proteins yields novel products of 4-(methylthio) butanal, 3-methylbutanal (isovaleraldehyde) and indole-3-acetaldehyde from methionine, leucine and tryptophan respectively. A comparative biochemical analysis of the *Medicago truncatula* and *Cicer arietinum* enzymes against two previously characterized SDC-like enzymes further emphasizes the unusual substrate specificity and activity of these novel aldehyde synthases. Due to the strong substrate preference towards phenylalanine, it is likely that both enzymes function as phenylacetaldehyde synthesis in vivo. However, due to their significant sequence divergence and unusual substrate promiscuity these enzymes are functionally and evolutionary divergent from canonical phenylacetaldehyde synthesis enzymes. This work further elaborates on the functional complexity of plant type II PLP decarboxylases and their roles in secondary metabolite biosynthesis.

**Electronic supplementary material:**

The online version of this article (doi:10.1186/s12870-014-0247-x) contains supplementary material, which is available to authorized users.

## Background

Biochemical characterization of a serine decarboxylase (SDC) was first established in *Arabidopsis thaliana* (AtSDC) [1]. It was proposed that the enzyme played a major role in choline synthesis by producing ethanolamine, a major intermediate for choline production [2,3]. Ethanolamine is also a precursor of phosphatidylethanolamine (PE) and phosphatidylcholine (PC); both PE and PC are major phospholipids in eukaryotic membranes [4-6]. The importance of this SDC in *A. thaliana* development has been demonstrated through the investigation of the AtSDC deficient mutant. A T-DNA insertion in the single *A. thaliana* SDC gene showed developmental defects, including necrotic leaf lesions, multiple inflorescences and flower sterility [7].

The functional characterization of the AtSDC enzyme provided a template to predict similar functions of homologous proteins based on their sequence homology without extensive biochemical verification [1,7]. Indeed, a GenBank search revealed a number of uncharacterized plant SDC-like sequences annotated as SDC proteins or SDC-like proteins. Additionally, it was noticed that many SDC-like proteins also were annotated as histidine decarboxylase (HDC)-like proteins. A literature search revealed that the HDC annotation in plants occurred from the cloning of a truncated tomato ortholog with high similarity towards bacterial HDC sequences [8]. However, a study of two plant HDC-like enzymes demonstrated their strict decarboxylation activity to serine with no measurable activity towards histidine [1]. Based upon the functional study of these enzymes, the authors suggest that all plant sequences annotated as HDC likely function as SDCs [1]. In our database search, we also found that some individual *Solanum lycopersicum* SDC-like sequences were annotated as aromatic amino acid decarboxylase (AAAD). Biochemical analysis of one of these SDC-like AAADs sequences (SlAAAD) demonstrated significant decarboxylation activity to tyrosine and phenylalanine [9]. Despite displaying aromatic amino acid decarboxylation activity, these tomato enzymes have limited homology to other characterized plant AAADs (10-15% identity) [10-13]. Rather, these tomato AAADs share significantly increased homology to the characterized plant AtSDCs (57% identity) [1].

Due to the extensive sequence identity between the functionally different SlAAAD and AtSDC enzymes, it was presumed that these enzymes might share some overlap in substrate specificity. To clarify the biochemical activity of both of these SDC-like sequences, we assessed the AtSDC enzyme for activity towards aromatic amino acids and the SlAAAD enzyme for activity towards serine. Additionally, we expressed and characterized two previously uninvestigated SDC-like enzymes from *Medicago truncatula* and *Cicer arietinum* in an effort to identify overlap in SlAAAD and AtSDC substrate selectivity. Our study of these uncharacterized SDC-like proteins led to an interesting discovery. Our data clearly show that both the *M. truncatula* and *C. arietinum* proteins function as acetaldehyde synthases with substrate preferences for bulky hydrophobic amino acids. In this report, we provide data that describe the substrate specificity, catalytic reaction and kinetic properties of these recombinant enzymes. This study of the novel activity of the *M. truncatula* and *C. arietinum* acetaldehyde synthases provides insights for a better understanding of the functional evolution of plant type II pyridoxal 5′-phosphate decarboxylases.

## Results

### Qualitative analysis of AtSDC and SlAAAD activities

Initially, our interest in SDC-like enzymes was aroused from the report of the unusual tomato SDS-like SlAAADs [9]. Although SDCs and AAADs are proposed to have a common evolutionary ancestor, significant evolutionary divergence has occurred between these two groups resulting in limited sequence conservation. While individual enzymes within each group (AAADs and SDCs) retain high sequence identity (typically greater than 50%), enzymes between these related groups maintain significantly reduced identity (typically lower than 15%). Therefore, the high sequence identity (57%) between the aromatic amino acid decarboxylating SlAAADs and serine decarboxylating AtSDC is quite unusual. In fact, the extensively shared identity of these enzymes led us to believe that there were likely overlaps in substrate specificity. The original characterization of SlAAAD did not test serine as a substrate while the original report of AtSDC did not examine if phenylalanine serves as a potential substrate [1,9]. To investigate their true substrate profiles, both the AtSDC and the SlAAAD were expressed, purified and subjected to decarboxylation activity assays. Analysis of the AtSDCs substrate preference confirmed the results of the original report [1]. AtSDC only has activity towards serine with no measurable decarboxylation of histidine, dopa, tyrosine, phenylalanine, tryptophan or glutamate (Additional file 1: Figure S1). An identical SlAAAD decarboxylation assay demonstrated activity towards tyrosine, dopa, phenylalanine and tryptophan with no activity towards serine, histidine or glutamate (Additional file 1: Figure S2). These results confirmed the separate functions of these highly homolgous enzymes.

### Characterization of MtAAS

**Figure 1:**
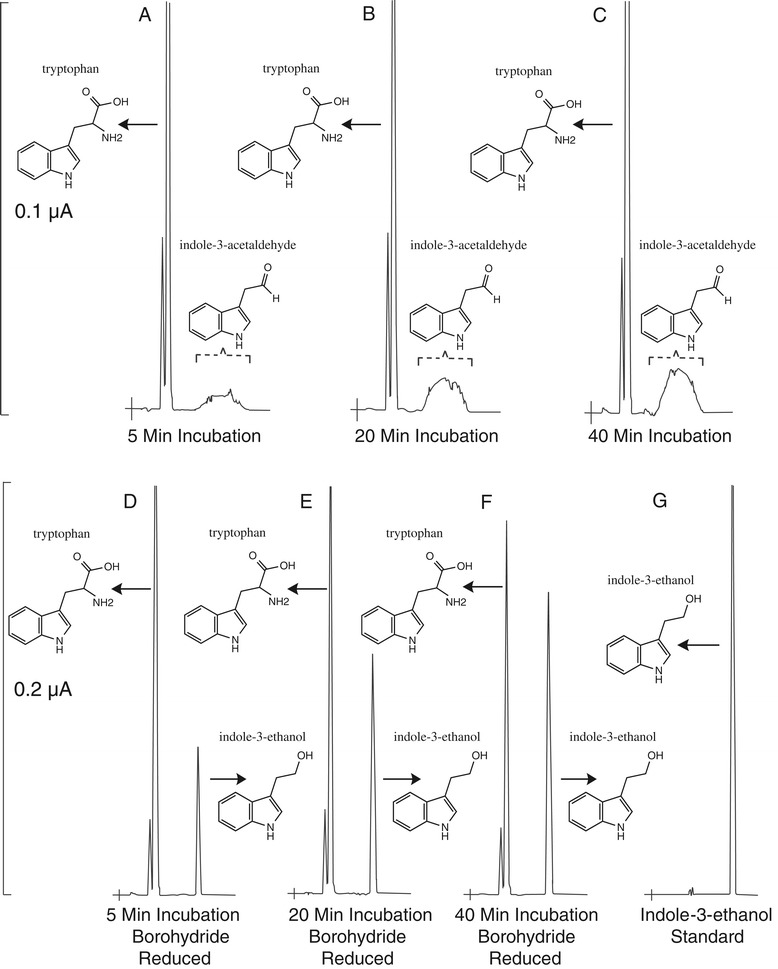
**HPLC-EC detection of indole-3-acetaldehyde generated in MtAAS and tryptophan reaction mixtures.** (Chromatograms **A-F**) Y-axis represents the output in microamps and the x-axis represents retention time. Chromatograms **(A-C)** illustrate the indole-3-acetaldehyde (the major broad peak) formed in MtAAS and tryptophan reaction mixtures after 5 min, 20 min and 40 min incubation, respectively. Chromatograms **(D-F)** illustrate the indole-3-ethanol (tryptophol) formed in borohydride reduced MtAAS and tryptophan reaction mixtures after 5 min, 20 min and 40 min incubation, respectively. Chromatogram **(G)** shows the detection of authentic indole-3-ethanol standard.

**Figure 2:**
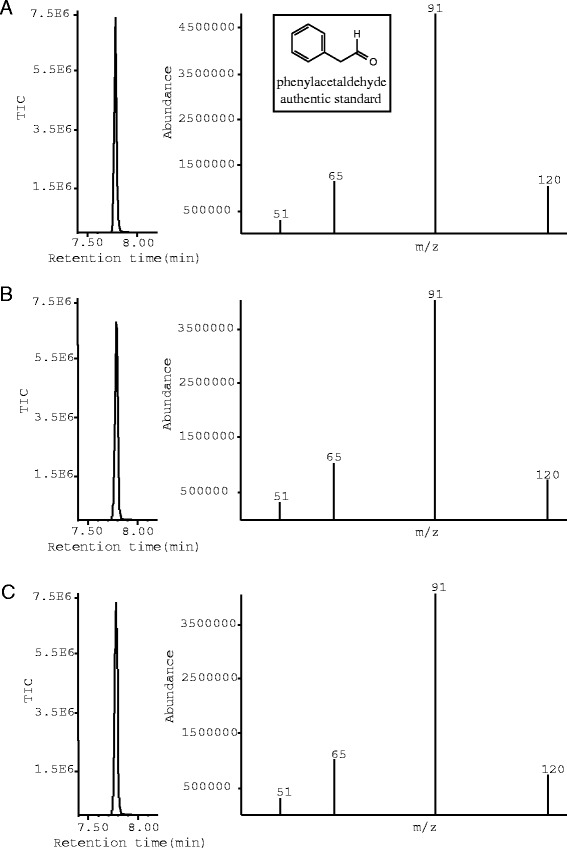
**GCMS analysis of authentic phenylacetaldehyde and MtAAS/CaAAS phenylalanine reaction products. (A)** illustrates the elution and select ion monitoring of authentic phenylacetaldehyde. **(B)** illustrates the elution and select ion monitoring of the enzymatic product generated from MtAAS and phenylalanine. **(C)** illustrates the elution and select ion monitoring of the enzymatic product generated from MtAAS and phenylalanine.

In an effort to evaluate biophysical characteristics capable of differentiating these functionally divergent enzymes, we have cloned, expressed, and purified a SDC-like enzyme from *Medicago truncatula* (MtAAS) and a SDC-like enzyme from *Cicer arietinum* (CaAAS)*.* MtAAS and CaAAS were initially assayed using known group II amino acid decarboxylase substrates (serine, histidine, glutamate, tyrosine, dopa, tryptophan, and 5-hydroxytryptophan) via an high-performance liquid chromatography electrochemical detection assay (HPLC-EC) [10,13,14]. Despite demonstrating no measurable amine product formation from any of the tested substrates, a broad peak was detected in the tryptophan reaction mixtures for each enzyme. The peak dimension increased proportionally as the incubation time increased (Figure 1A-C), indicating that the broad peak corresponds to the reaction product. The product peak in the recombinant enzymes and tryptophan reaction mixtures appeared to be an aromatic acetaldehyde based on its similar chromatographic behavior to previously investigated aromatic acetaldehydes [13,15,16]. This acetaldehyde-like peak suggested that the MtAAS and CaAAS enzymes might function as aromatic aldehyde synthases rather than a SDC. Aldehydes can be reduced to their corresponding alcohol by borohydride [13,15]. When the recombinant protein and tryptophan reaction mixtures were treated with NaBH_4_ prior to HPLC-ED analysis, the broad product peak (Figure 1A-C) was converted to a sharp peak (Figure 1D-F). The sharp peak, detected in the borohydride-treated reaction mixture, had identical retention time as authentic indole-3-ethanol under the same conditions of HPLC-EC analysis and coeluted with the standard at different mobile phase conditions during HPLC-EC analysis (Figure 1G). Comparison of the chromatographic behavior of the product and authentic tryptamine suggests that these enzymes function as a novel aldehyde synthases and not as a decarboxylases (Additional file 1: Figure S3). Gas chromatography mass spectrometry (GCMS) was subsequently used to further establish the activities of these enzymes. The identical elution time and fragmentation pattern of phenylalanine-enzyme products and an authentic phenylacetaldehyde standard demonstrates MtAAS and CaAASs roles in acetaldehyde production (Figure 2).

**Figure 3:**
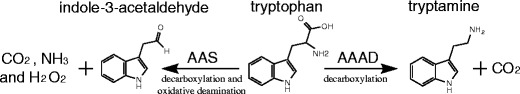
**Relative activities of aromatic amino acid decarboxylase (AAAD) and aromatic acetaldehyde synthases (AAS).**

To investigate the substrate specificity of these novel aldehyde synthases (AASs) a peroxide assay was performed against each of the 20-proteinogenic amino acids (plus 5-hydroxytryptophan and dopa). Enzyme reaction mixtures were then assayed through the use of the Pierce® Quantitative Peroxide Assay Kit. AASs catalyze a rather complicated decarboxylation-oxidative deamination process of aromatic amino acids, leading to the production of aromatic acetaldehydes, CO_2_, ammonia, and hydrogen peroxide rather than the AAAD derived arylalkylamines and CO_2_ (Figure 3) [13,15,17,18]. Therefore, the production of hydrogen peroxide can be used as a marker to further differentiating AAS and AAAD enzymatic activities. Results for both MtAAS and CaAAS demonstrated very minimal acetaldehyde synthase activity to the majority of tested substrates and significant acetaldehyde synthase activity towards several bulky, non-polar and hydrophobic amino acids (phenylalanine, methionine, leucine and tryptophan).

### Kinetic properties of MtAAS and CaAAS

Next, amino acids demonstrating significant specific activity were used in a full kinetic study of the MtAAS and CaAAS enzymes. The profile of kinetically characterized substrates includes phenylalanine, methionine, leucine and tryptophan. Results demonstrated that the aforementioned amino acids function well as substrates (Table 1) (Additional file 1: Figure S4–S5). All active substrates share similar biophysical characteristics (bulky, non polar, and hydrophobic). This substrate promiscuity is highly atypical of characterized AASs [15,17,18].

**Table 1:** **Kinetic parameters of MtAAS and CaAAS enzymes**

**Enzyme**	**Substrate**	**kcat**	**km**	**kcat/Km**
		**(sec** ^**−1**^ **)**	**(mM)**	**(sec** ^**−1**^ **mM)**
MtAAS	Phenylalanine	0.358±0.005	0.02±0.01	17.90±2.29
MtAAS	Methionine	0.144±0.006	1.90±0.20	0.08±0.01
MtAAS	Tryptophan	0.125±0.008	1.70±0.30	0.07±0.01
MtAAS	Leucine	0.197±0.005	7.60±0.70	0.03±0.01
CaAAS	Phenylalanine	0.595±0.009	0.09±0.01	6.60±0.65
CaAAS	Methionine	0.351±0.012	1.62±0.20	0.22±0.01
CaAAS	Tryptophan	0.187±0.016	2.80±0.70	0.07±0.1
CaAAS	Leucine	0.467±0.010	4.60±0.40	0.10±0.01

Values represent means SE (n = 3).

### Comparison of substrate promiscuity

Results from the kinetic characterization of MtAAS and CaAAS elaborated on the unusual activity and substrate specificity of the enzymes. To emphasize the promiscuous nature of these AASs, we have tested their preferred substrates against the homologous AtSDC and the SlAAAD enzymes. Literature searches in addition to our own analysis indicate that the AtSDC and SlAAAD enzymes both maintain stringent substrate specificity [1,9]. The recombinantly characterized AtSDCs only displayed activity towards serine while the recombinantly characterized SlAAAD catalyzed the decarboxylation of aromatic substrates (phenylalanine, tyrosine, dopa and tryptophan). An HPLC-EC assay of SlAAAD and AtSDC using MtAASs preferred substrates (phenylalanine, methionine, leucine and tryptophan) further verify the previous reported AtSDC and SlAAAD activity. Results indicate that AtSDC lacks measurable activity towards any of the MtAAS and CaAAS substrates while the SlAAAD lacks activity towards leucine and methionine. In addition to divergent substrate specificities, an AAS hydrogen peroxide assay of AtSDC and SlAAAD towards their preferred substrates (serine and tyrosine respectively) demonstrated no peroxide production (Additional file 1: Figure S6). The lack of AAS activity and limited substrate profile of AtSDC and SlAAAD serve to highlight the unusual nature of the recombinantly characterized promiscuous MtAAS and CaAAS enzymes.

## Discussion

Type II pyridoxal 5′-phosphate (PLP)-dependent decarboxylases are a group of enzymes with important roles in amino acid metabolism. This group of enzymes has undergone functional evolution from a shared ancient evolutionary origin to generate a selection of subfamilies with stringent substrate selectivity’s [14]. Plant type II PLP decarboxylases include aromatic amino acid decarboxylases (AAADs), serine decarboxylases (SDCs) and glutamate decarboxylases (GDCs). Plant SDSs catalyze the decarboxylation of serine to ethanolamine [1], GDCs catalyze the decarboxylation of glutamate to γ-aminobutyric acid (GABA) [19] and AAADs catalyze the decarboxylation of aromatic amino acids to generate aromatic arylalkylamines [10-12]. Based on their respective substrate specificities each group is responsible for the biosynthesis of unique products [1,10,19]. Although all plant type II PLP decarboxylases have evolved from a common evolutionary ancestor, significant evolutionary divergence has occurred resulting in limited sequence conservation [14]. While individual enzymes within each group (AAADs, SDCs, and GDCs) maintain high identity (typically greater than 50%), enzymes between these related groups maintain significantly reduced identity (typically lower than 15%). For example, the characterized *Arabidopsis thaliana* enzymes from each class demonstrate 9% identity between GDC (NP_*197235) and SDC (NP*_175036), 5% identity between GDC and AAAD (NP_001078461), and 14% identity between SDC and AAAD.

Unlike other plant type II PLP decarboxylases, plant AAADs have undergone additional functional evolution resulting in multiple paralogs with divergent functions [10]. Plant AAAD subfamilies include tryptophan decarboxylases (TDCs) [12], tyrosine decarboxylases (TyDCs) [11] and aromatic acetaldehyde synthases (AASs) [17]. TDCs and TyDCs catalyze the decarboxylation of indolic and phenolic amino acids respectively to generate their corresponding aromatic arylalkylamines while AAS catalyze a more involved decarboxylation/oxidative deamination reaction to generate aromatic acetaldehydes from their phenolic amino acid substrates. Although this functional divergence is well documented within plant AAADs [10], there has been no reports of similar divergence within plant SDCs or GDCs.

In this study we have investigated plant SDC-like enzymes in an effort to evaluate their functional divergence. To gain additional insight into variations in substrate selectivity, we have analyzed two SDC-like enzymes from *Medicago truncatula* and *Cicer arietinum*. Activity assays and a full kinetic characterization of the MtAAS and CaAAS demonstrated novel aldehyde synthase enzymes with activity towards phenylalanine, methionine, leucine and tryptophan. These SDC-like enzymes are capable of generating phenylacetaldehyde, 4-(methylthio) butanal, 3-methylbutanal (isovaleraldehyde) and indole-3-acetaldehyde from phenylalanine, methionine, leucine and tryptophan respectively. Judging by the respective k_cat_/K_m_ values of the MtAAS and CaAAS substrates in addition to the previous characterization of phenylalanine decarboxylation and oxidative deamination enzymes [17,18], it is likely that MtAAS and CaAAS function as a phenylacetaldehyde synthases (PAAS) for the *in vivo* production of phenylacetaldehyde (a floral volatile [20-22]). Despite the obvious preference for phenylalanine as a substrate, tryptophan, methionine and leucine have specificity constants comparable to other recombinantly characterized PAAS enzymes. For example the k_cat_/K_m_ for the petunia PAAS and the Arabidopsis PAAS towards phenylalanine are k_cat_/K_m_ 0.678 sec^−1^ mM^−1^ and k_cat_/K_m_ 0.012 sec^−1^ mM^−1^ respectively [17,18]. The physiologically relevant k_cat_/K_m_ values of MtAAS and CaAAS towards tryptophan, methionine and leucine indicate that these substrates may be catalyzed to product formation *in vivo.*

**Figure 4:**
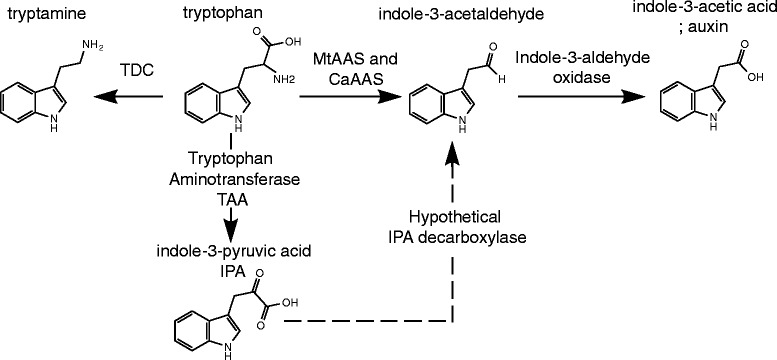
**Intersection of the MtAAS and CaAAS enzymes and the proposed tryptophan dependent indole-3-pyruvic acid auxin biosynthetic pathway.**

If phenylalanine were indeed the preferred physiological substrate of MtAAS and CaAAS, then one might ask, why would these enzymes demonstrate significant and unusual activity towards other biophysically similar amino acids. Two potential explanations occur to us. First, these enzymes do indeed use these amino acid substrates for the production of evolutionary useful compounds. Second, these enzymes have recently (from an evolutionary perspective) diverged from an SDC and are currently in the process of evolving and tuning the enzymes specificity towards phenylalanine. To analyze the first explanation, we have performed literature searches in an effort to find examples of 4-(methylthio) butanal, 3-methylbutanal (isovaleraldehyde) and indole-3-acetaldehyde product formation. Although 4-(methylthio) butanal and 3-methylbutanal (isovaleraldehyde) proved to be unknown enzyme products, there have been many references regarding the production of indole-3-acetaldehyde [13,23,24]. Indole-3-acetaldehyde is a proposed intermediate in the original tryptophan dependent indole-3-pyruvic acid (IPA) auxin biosynthetic pathway [23,24]. Although many references suggest indole-3-acetaldehyde as an auxin intermediate, a full indole-3-acetaldehyde dependent biosynthetic pathway has not been verified. Despite the identification of plant aldehyde oxidases capable of catalyzing the conversion of indole-3-acetaldehyde to indole-3-acetic acid (IAA) there have thus far been no enzymes capable of generating indole-3-acetaldehyde [25,26]. Interestingly, the decarboxylation and oxidative deamination of tryptophan via MtAAS or CaAAS is capable of performing this very function. Therefore, it is reasonable to suggest this enzyme could be a possible link in the biosynthesis of auxin (Figure 4).

The second explanation regarding unusual product formation of MtAAS suggests that 4-(methylthio) butanal, 3-methylbutanal (isovaleraldehyde) and indole-3-acetaldehyde are unintended byproducts in phenylacetaldehyde production. Such catalytic promiscuity appears to be a common consequence of secondary metabolite biosynthetic enzymes [27-29]. This mechanistic elasticity of secondary metabolite biosynthetic enzymes often results in diminished catalytic efficiency with greater substrate permissiveness [27-29]. Although unintended chemistry and product formation may occur, the synthesis of a product that confers a fitness advantage will still drive the proliferation of the gene. Individual secondary metabolite biosynthetic enzymes do not require exact substrate specificity or chemistry; they only require the synthesis of a useful compound to be maintained in the population. Moreover, such promiscuous substrate specificity may enable individual enzymes to play multiple physiological roles. One of the minor products may subsequently grant a reproductive advantage as the organism is exposed to a fluctuating environment.

## Conclusions

This work has identified functional divergence of plant SDC-like enzymes. Through enzymatic divergence some SDC-like enzyme have developed novel substrate preferences and chemistry to generate an altered profile of products. In this study, we have characterized two AAS enzyme with unusual aldehyde synthase activity towards phenylalanine, tryptophan, methionine, and leucine. Although it is likely that these enzymes functions as a PAASs for the production of flower volatiles, additional product aldehyde formation opens the options for alternative physiological roles.

## Methods

### Reagents

Alanine, arginine, asparagine, aspartic acid, cysteine, glutamine, glutamic acid, glycine, histidine, isoleucine, leucine, lysine, methionine, phenylalanine, proline, serine, threonine, tryptophan, 5-hydroxytryptophan, tyrosine, valine, dopa, phenylacetaldehyde, pyridoxal 5-phosphate, formic acid, phthaldialdehyde, hydrogen peroxide solution and acetonitrile were purchased from Sigma (St. Louis, MO). The IMPACT-CN protein expression system was purchased from New England Biolabs (Ipswich, MA).

### Preparation of the recombinant proteins

*M. truncatula* cDNA and *S. lycopersicum* cDNA were obtained through Dr. Jiangqi Wen at the Noble Institute and Dr. Richard Veilleux from the Virginia Tech horticulture department, respectively. *C. acuminata* seeds were obtained through Bountiful Gardens. *C. acuminata* seeds were germinated in Sunshine Pro Premium potting soil and were grown under a 16 h photoperiod at 23°C. at 100 microeinsteins. Total RNA was isolated from whole plants (12 weeks) Ambion mirVana™ miRNA Isolation Kit. RNA samples were subsequently DNase-treated using Ambion TURBO DNA-free™ Kit. cDNAs were produced using Invitrogen™ SuperScript™ III First-Strand Synthesis System for RT-PCR. *A. thaliana* cDNA was prepared as previously described [13]. Primer pairs were synthesized and used for the amplification of the *M. truncatula* [GenBank:XP_003592128] MtAAS gene, the *C. arietinum* [GenBank:XP_004496485] CaAAS gene, the *S. lycopersicum* NP_001233845 SlAAAD gene and the *Arabidopsis thaliana* NP_175036 AtSDC gene (Additional file 1: Table S1).

The resulting PCR products were ligated into the pTYB12 IMPACT-CN bacterial expression plasmid. DNA sequencing was utilized to verify the sequences and frame of each cDNA insert. Transformed bacterial colonies were selected and used for large-scale expression of individual recombinant proteins. Bacterial cells were cultured at 37°C. After induction with 0.15 mM IPTG, the cells were cultured at 15°C for 24 hrs. The soluble fusion proteins were applied to a column packed with chitin beads and subsequently hydrolyzed under reducing conditions. The affinity purification resulted in the isolation of each individual recombinant protein at about 85% purity. Further purifications of the recombinant proteins were achieved by Mono-Q and gel filtration chromatographies (greater than 95% purity). Purity of the recombinant proteins was evaluated by SDS-PAGE. Purified recombinant enzymes were concentrated to 5 mg/ml protein in 20 mM HEPES (pH 7.5), containing 5 mM PLP using a Centricon YM-50 concentrator (Millipore). Using bovine serum albumin as a standard, purified recombinant proteins concentrations were determined by a Bio-Rad protein assay kit (Hercules, CA).

### MtAAS and CaAAS activity assays

Typical reaction mixtures of 50 μl, containing 25 μg of MtAAS recombinant enzyme and 5 mM substrate (20 proteinogenic amino acids plus 5-hydroxytryptophan and dopa) were prepared in 20 mM HEPES (pH 7.5) and incubated at 25°C in a water bath. The reactions were stopped after 20 minutes through the addition of 50 μl of 0.8 M formic acid. Supernatants of the reaction mixtures, obtained by centrifugation, were analyzed with (Aqueous) Pierce Quantitative Peroxide Assay Kit to determine AAS activity. Tryptophan reaction mixtures were also analyzed by HPLC-EC. 50 μl reactions containing 25 μg of recombinant enzyme and 8 mM tryptophan were prepared in 20 mM HEPES (pH 7.5) and incubated at 25°C in a water bath for 5, 20 or 40 minutes. The reactions were stopped through the addition of 200 μl of 0.8 M formic acid or with 200 μl of borohydride saturated ethanol solution. Separation was achieved through a 50 mM potassium phosphate isocratic running buffer (pH 4.0) with 0.5 mM octyl sulfate and 45% acetonitrile. Indole-3-ethanol was verified though the comparison of authentic standards under identical chromatography conditions.

### MtAAS and CaAAS GCMS product verification

To verify the identity of the MtAAS and CaAAS enzymatic products, reaction mixtures containing phenylalanine and recombinant enzymes were analyzed by GCMS. Reaction mixtures of 500 μl, containing 50 μg of recombinant enzyme and 10 mM phenylalanine were prepared in 20 mM HEPES (pH 7.5) and incubated at 25°C in a water bath for 20 min. Reactions were stopped by mixing an equal volume of 0.8 M formic acid. Prior to GCMS analysis, products were extracted with 100ul of ethyl acetate. 1 μl samples were analyzed by an Agilent Technologies 7890B GC and a 5977A MS. Separation was achieved with a 250°C injection port, an oven temperature range of 45–185°C and a HP 5MS column. Identification of phenylacetaldehyde from the MtAAS and CaAAS reactions was based on their retention time and spectra in comparison with those produced from 500 μM authentic phenylacetaldehyde at identical analytic conditions.

### Kinetic analysis

Initial MtAAS and CaAAS activity assays indicated that at high substrate concentrations (5 mM) several hydrophobic amino acids demonstrated significant activity. To determine the binding affinity and reaction velocity the kinetic parameters of the enzyme were analyzed using leucine, methionine, tryptophan, and phenylalanine. Reaction mixtures of 50 μl containing 5 μg of recombinant protein and varying concentration of substrate (0.0025 – 45 mM; depending on the solubility of individual amino acids) were prepared in 20 mM HEPES (pH 7.5) and incubated at 25°C. An equal volume of 0.8 M formic acid was added to each reaction mixtures after 5 min of incubation and analyzed using the Pierce® Quantitative Peroxide Assay Kit. Product formation was compared to standards generated from 30% (w/w) hydrogen peroxide solution. Kinetic data points were performed in triplicate and kinetic values were evaluated by hyperbolic regression.

### MtAAS CaAAS AtSDC and SlAAAD activity comparisons

HPLC-EC was used to further illustrate the unusual substrate range of MtAAS and CaAAS enzymes. Purified AtSDC and SlAAAD recombinant enzymes were assayed against the preferred substrates of MtAASs and CaAAS (phenylalanine, methionine, leucine and tryptophan) in addition to other common type II PLP decarboxylase substrates (histidine, serine, glutamate, dopa and tyrosine). Reaction mixtures of 50 μl containing 15 μg of recombinant protein and 5 mM substrate were prepared in 20 mM HEPES (pH 7.5) and stopped with an equal volume of 0.8 M formic acid after 10 min of incubation at 25°C. The products (besides tryptophan, 5-hydroxytryptophan, dopa, tyrosine, reactions) were then derivatized with OPA-thiol reagent (to convent amine to electrochemically active compound). Various isocratic running buffers consisting of 50 mM phosphate buffer pH 4.0, 0.5 mM octyl sulfate and a acetonitrile range of 40-55% were used for the OPA-thiol characterization. An isocratic running buffer consisting of 50 mM phosphate buffer pH 4.3, 28% acetonitrile, and 0.5 mM octyl sulfate was used for the tryptamine products. An isocratic running buffer consisting of 50 mM phosphate buffer pH 4.3, 18% acetonitrile, and 0.5 mM octyl sulfate was used for the dopamine and tyramine products.

## Additional file

Additional file 1:
**Supplementary information.**

## Abbreviations

PLP: pyridoxal 5′-phosphate
AAADs: aromatic amino acid decarboxylases
SDCs: serine decarboxylases GDCs, glutamate decarboxylases
GABA: glutamate to γ-aminobutyric acid
TDCs: tryptophan decarboxylases
TyDCs: tyrosine decarboxylases
AASs: aromatic acetaldehyde synthases
HPLC: high-performance liquid chromatography
HPLC-EC: high-performance liquid chromatography electrochemical detection
GCMS: gas chromatography mass spectrometry
