# Supplementary material for: Diverse functional evolution of serine decarboxylases: identification of two novel acetaldehyde synthases that uses hydrophobic amino acids as substrates
Source: BMC Plant Biol. 2014 Sep 18;14:247. doi: 10.1186/s12870-014-0247-x (PMC4177580; doi:10.1186/s12870-014-0247-x)
Supplement: Additional file 1: — Supplementary information. [file 12870_2014_247_MOESM1_ESM.docx]

**Supplemental Figure 1.** HPLC-EC analysis of SlAAAD activity with dopa, tyrosine, phenylalanine and tryptophan as substrates. Y-axis represents the output in microamps and the x-axis represents retention time. Reaction mixtures of 50 μl containing 15 μg of SlAAAD and 5 mM of substrate were incubated at 25 ^o^C and their reaction was stop at 10 min after incubation by adding an equal volume of 0.8 M of formic acid into the reaction mixture. After stopping the reaction with formic acid, the phenylalanine reaction mixture was treated o-phthaldialdehyde / 2-mercaptoethanol reagent to generate an electrochemically active conjugate. The mixtures were centrifuged for 5 min at 14,000g and supernatants were injected for HPLC-EC analysis. Chromatogram (A) illustrates the accumulation of dopamine in a SlAAAD and dopa reaction mixture. Chromatogram (B) illustrates the accumulation of tyramine in a SlAAAD and tyrosine reaction mixture. Chromatogram (C) illustrates the accumulation of phenylethylamine in a SlAAAD and phenylalanine reaction mixture (samples were derivatized with phthalaldehyde) Chromatogram (D) illustrates the accumulation of tryptamine in a SlAAAD and tryptophan reaction mixture. Various isocratic running buffers consisting of 50 mM phosphate buffer pH 4.0, 0.5 mM octyl sulfate and a acetonitrile range of 18-55% were used for the characterization.

**Supplemental Figure 2.** HPLC-EC analysis of AtSDC activity with serine as a substrate. Y-axis represents the output in microamps and the x-axis represents retention time. The reaction mixture of 50 μl containing 15 μg of SlAAAD and 5 mM of serine was incubated at 25 ^o^C and stopped after 10 min of incubation by adding an equal volume of 0.8 M formic acid. Next, the reaction mixture was treated o-phthaldialdehyde / 2-mercaptoethanol reagent to generate an electrochemically active conjugate. The mixture was centrifuged for 5 min at 14,000g and supernatant was injected for HPLC-EC analysis. The chromatogram illustrates the accumulation of ethanolamine in an AtSDC and serine reaction mixture. An isocratic running buffer consisting of 50 mM phosphate buffer pH 4.0, 0.5 mM octyl sulfate and 40% acetonitrile was used for the characterization.

**Supplemental Figure 3.** HPLC-EC analysis of MtAAS activity with tryptophan as a substrate. Y-axis represents the output in microamps and the x-axis represents retention time. The reaction mixture of 50 μl containing 15 μg of MtAAS and 5 mM of tryptophan was incubated at 25 ^o^C and stopped after 10 min of incubation by adding an equal volume of 0.8 M of 100% ethanol saturated with borohydride. The mixture was centrifuged for 5 min at 14,000g and supernatant was injected for HPLC-EC analysis. Chromatogram (A) illustrates the accumulation of indole-3-acetaldehyde (subsequently reduced to indole-3-ethanol via borohydride) in an MtAAS and tryptophan reaction mixture. Chromatogram (B) illustrates a tryptophan and tryptamine standard. An isocratic running buffer consisting of 50 mM phosphate buffer pH 4.0, 0.5 mM octyl sulfate and 28% acetonitrile was used for the characterizations.

**Supplemental Figure 4** Kinetic regression curves for the MtAAS enzyme and (A) phenylalanine, (B) tryptophan, (C) Methionine and (D) leucine.

**­**

**Supplemental Figure 5** Kinetic regression curves for the CaAAS enzyme and (A) phenylalanine, (B) tryptophan, (C) Methionine and (D) leucine.

**Supplemental Figure 6**. Analysis of hydrogen peroxide generated from MtAAS, AtSDC and SlAAAD. The preferred substrate was used for each enzyme. Phenylalanine was used for MtAAS, serine was used for AtSDC and tyrosine was used for SlAAAD. Reaction mixtures of 0.2 ml containing 5 mM substrate and 1ug of recombinant enzyme were prepared in 20 mM HEPES (pH 7.5). The reaction mixtures were incubated at 25 °C. At each 1-min interval, 20 μl of reaction mixture was withdrawn and mixed into 200 μl of Pierce peroxide assay reagents solution. The MtAAS, AtSDC and SlAAAD curves illustrate the amount of H_2_O_2_accumulated

in 20 μl of reaction mixtures at a 1–8-min incubation periods. Product formation from AtSDC and SlAAAD reaction mixtures are displayed on separate graphs to maintain figure clarity.

**Supplemental Table 1.**
